# SHARED: An International Collaboration to Unravel Hepatitis C Resistance

**DOI:** 10.3390/v13081580

**Published:** 2021-08-10

**Authors:** Anita Y.M. Howe, Francesca Ceccherini-Silberstein, Julia Dietz, Stephanie Popping, Jason Grebely, Chaturaka Rodrigo, Johan Lennerstrand, Mark W. Douglas, Milosz Parczewsk, P. Richard Harrigan, Jean-Michel Pawlotsky, Federico Garcia, SHARED Collaborators

**Affiliations:** 1 British Columbia Centre for Disease Control, Vancouver, BC V5Z 4R4, Canada; 2 Department of Experimental Medicine, University of Rome Tor Vergata, 00133 Rome, Italy; ceccherini@med.uniroma2.it; 3 Department of Internal Medicine 1, University Hospital, Goethe University, 65926 Frankfurt, Germany; julia.dietz@em.uni-frankfurt.de; 4 Erasmus Medical Center, 3015 CN Rotterdam, The Netherlands; s.popping@erasmusmc.nl; 5 The Kirby Institute, UNSW Sydney, Sydney, NSW 2006, Australia; jgrebely@kirby.unsw.edu.au; 6 Department of Pathology, University of New South Wales, Sydney, NSW 2006, Australia; c.rodrigo@unsw.edu.au; 7 Department of Medical Sciences, Clinical Microbiology, Uppsala University, 75121 Uppsala, Sweden; johan.lennerstrand@medsci.uu.se; 8 Storr Liver Centre, The Westmead Institute for Medical Research, The University of Sydney at Westmead Hospital, Westmead, NSW 2145, Australia; mark.douglas@sydney.edu.au; 9 Department of Infectious Tropical Diseases and Immune Deficiency, Pomeranian Medical University in Szczecin, 70-507 Szczecin, Poland; mparczewski@yahoo.co.uk; 10 Department of Medicine, University of British Columbia, Vancouver, BC V5Z 4R4, Canada; richard.harrigan@ubc.ca; 11 National Reference Center for Viral Hepatitis B, C and D, Department of Virology, Henri Mondor Hospital & INSERM U955, 94000 Créteil, France; jean-michel.pawlotsky@aphp.fr; 12 Microbiology Department, University Hospital San Cecilio, Instituto de Investigacion Ibs.Granada, 18012 Granada, Spain; fegarcia@ugr.es

## 1. Is Resistance Surveillance Needed in the Era of DAAs?

The advent of direct-acting antivirals (DAAs) has transformed the treatment landscape of hepatitis C. To date, over 95% of the hepatitis C virus (HCV)-infected patients who received DAA treatments have achieved sustained viral response (SVR, or “cure”) [1,2]. Despite this successful therapeutic intervention, no vaccine is available for HCV. In 2016, the World Health Organization proposed ambitious global targets to eliminate viral hepatitis by 2030 [3]. In this proposal, HCV elimination relies on the “treatment as prevention” approach. One potential barrier to this approach is the presence of drug-resistant viruses. The occurrence of resistance (either natural or after failure) can limit treatment effectiveness, and these drug-resistant viruses can then be transmitted onward to others.

The prevalence of clinically relevant resistance-associated substitutions (RAS) in natural isolates varies among DAAs and genotypes (GTs), ranging from 2–25% in NS3 to 4–12% in NS5A, depending on the geographic region [4]. Although the effect of baseline RAS on the overall success of DAA treatment is relatively small, numerous examples have demonstrated reduced response rates in the presence of RAS. The presence of Q80K in NS3 resulted in decreased SVR among individuals treated with simeprevir–sofosbuvir [5]. Baseline NS5A RAS also reduced the efficacy of elbasvir–grazoprevir and ledipasvir–sofosbuvir in GT1a patients, requiring extended treatment duration [6]. The negative effects of specific baseline NS5A RAS on SVR were further manifested in GT3 patients with cirrhosis treated with ledipasvir–sofosbuvir, daclatasvir–sofosbuvir, velpatasvir–sofosbuvir, or glecaprevir–pibrentasvir [6,7]. Ongoing viral replication in the face of sub-optimal drug pressure during DAA treatment results in an enrichment of the pre-existing RAS or accumulation of additional RAS, which reduce drug susceptibility or enhance viral replicative fitness. Given that ~58 million individuals worldwide have active HCV infections [8], even 2–5% treatment failure rates would translate into a significant number of patients infected with resistant HCV. Transmission of resistance variants among men who have sex with men has been reported [9].

The combination of high response rates and the relatively small, regional, short-term studies of HCV therapy have led to inadequate evaluations of HCV resistance [10,11,12]. In studies where HCV drug resistance was assessed, no consistent criteria have been to determine which amino acid substitutions should be included in the analyses, with no standardization of analytical methods. Furthermore, most of the drug resistance studies have focused on GT1, and to a lesser extent GT3, which are most common in industrialized countries; information for GTs 2 and 4–6 is minimal. Genotypes 2 and 4–6 are endemic in resource-limited countries, where the prevalence of HCV infection is high and the data on drug resistance are severely lacking [13]. The scarcity of phenotypic data and the lack of clarity on resistance interpretation have significantly hampered the use of this information to guide clinical decision-making. For HIV, public resources such as the Stanford University HIV Drug Resistance Database have provided comprehensive information to correlate virus substitutions with treatments, phenotypes, and clinical outcomes of antiretroviral therapies [14]. Clinicians have widely used this information to design treatment strategies. A similar database does not exist for HCV.

Recent clinical studies revealed that patients infected with certain subtypes such as GT1l, 3b, 3g, 4r, 6u, and 6v had low treatment response rates [15,16,17,18]. Patients originating from Sub-Saharan Africa or South Asia are commonly infected with these “unusual” GT subtypes [18,19]. The term “unusual” refers to their low prevalence in high-income countries with access to therapy and their limited presence during clinical trials; these GT subtypes might be highly prevalent in other countries. At present, it is unclear which mechanism leads to lower response rates; however, initial data from in vivo and in vitro studies point to a high prevalence of inherent resistant polymorphisms in the natural isolates of these subtypes [20]. Novel substitutions and substitution patterns continue to emerge in real-world environments among those who fail therapy. A concerted effort to monitor these substitutions and their transmission is prudent to safeguard public health.

Preventing onward transmission and optimizing treatment success is paramount to achieving global HCV elimination. Epidemiological surveillance of drug resistance provides vital statistics on disease burden and aids in the assessment of treatment and prevention strategies. In the future, should there be a need to develop third or fourth generations of antivirals, resistance information will be crucial to optimize new chemical entities. Finally, information on HCV genetics and evolution can provide valuable insights into successful vaccine design. With DAAs being rolled out in different parts of the world and an inevitable selection of drug resistance, it is now time to set up large-scale data collection for drug resistance.

## 2. What Is SHARED?

**Figure 1:**
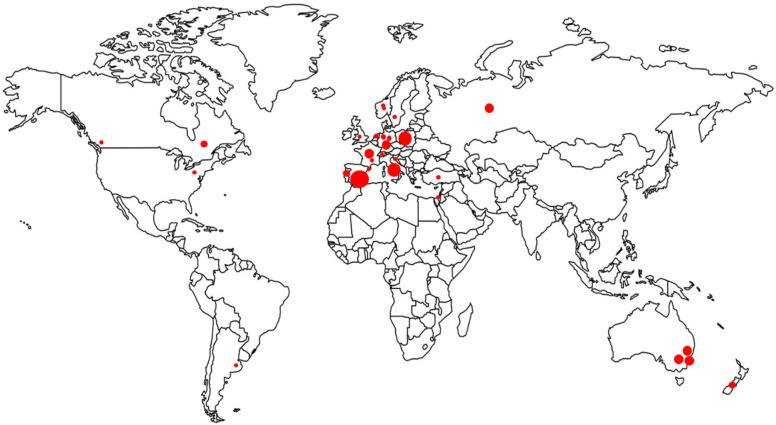
Geographic locations of participating centers and patient cohorts in SHARED. Each dot represents a participating center (local clinic or reference laboratory) where patient sample sequences were generated. For samples from randomized clinical trials or international consortia, the dots represent the participating countries. The size of the dot represents the relative number of samples (not to scale).

The Surveillance of Hepatitis C Antiviral Resistance, Epidemiology, and methoDologies (SHARED) is an international collaboration to create a sizeable merged dataset from cohorts of well-characterized HCV sequences linked with patient information, disease characteristics, regimen history, and treatment outcomes. This pooled dataset allows in-depth data analyses and generates insights on HCV antiviral resistance that are not possible from individual studies. SHARED has brought together researchers from Argentina, Australia, Austria, Canada, France, Germany, Israel, Italy, Luxemburg, Netherlands, Norway, New Zealand, Poland, Portugal, Romania, Russia, Slovenia, Spain, Switzerland, Sweden, Turkey, and the United States (Figure 1). The SHARED dataset came from nine HCV reference laboratories, six national patient cohorts, six randomized clinical trials, and one international network (HepCare) [21], including over 110 individual clinics and hospitals. As its name suggests, SHARED aims to: (i) characterize and harmonize interpretations of RAS; (ii) report on the real-word prevalence and transmission of HCV drug-resistant variants, with a particular interest in novel RAS and “unusual” HCV GT subtypes inherently resistant to DAAs; (iii) provide a platform to freely share assay protocols, software, and technologies for drug resistance and epidemiological evaluations.

The inception of SHARED dates back to 2016 at the Forum for Collaborative Research in Boston, USA, where clinicians and researchers across the globe unanimously identified the need to standardize HCV RAS evaluation and reporting. As there was no mandate for baseline resistance testing for HCV, it was decided that a database focused on treatment failures, including the “unusual” HCV GT subtypes, would provide the most value to the field. A small subset of baseline data from patients with SVR was also collected for comparison purposes. SHARED participation and data contribution were entirely voluntary; a small grant was obtained from Genome British Columbia and Merck to kick off the project. Contributing collaborators retain full ownership of their data and may submit data before or after publication. Members within the SHARED community are encouraged to use the pooled data for research and publications.

## 3. What Types of Data Are Collected?

The SHARED database comprises an enormous scope of data, including demographics (ethnicity, age, gender, country), transmission-related risk factors (injection or illicit drug use, sexual behavior, prison), clinical test results (liver enzymes, fibrosis scores, HIV–HBV co-infection, CD4 count, etc.), treatment information (treatment history, regimen, viral response), and virology data (HCV nucleotide and amino acid sequences, viral load, GT subtypes). Data were collected under medical or human research ethics committee-approved protocol at each respective collaborator’s site. De-identified data were sent through a secured file transfer protocol server to the coordinating center at the University of British Columbia, Canada, where data were curated, formatted, and stored in a relational database using MYSQL. The scheme of the SHARED database can be found at https://hcvdb.med.ubc.ca (accessed on 21 July 2021). Ethical approval for developing the SHARED database was granted by the University of British Columbia Research Ethics Board (H17-10589).

Between 2019 and 2021, data from 4911 patients from 22 countries were collected; these included over 10,300 HCV sequences from NS3, NS5A, and NS5B genes linked with anonymous demographic data, risk factors, clinical results, and treatment information (Table 1). The majority of the patients were infected with GT1 (63%) and GT3 (28%), the most prevalent epidemic strains circulating globally. Forty percent [40%] of these patients achieved SVR, while 60% experienced virologic failure. Most of the patients were treated with the first-generation DAAs with or without ribavirin—56% with NS5A inhibitor + sofosbuvir combinations, 19% with NS5A inhibitor + protease inhibitor combinations, 4% with protease inhibitor + sofosbuvir combinations, and 13% with triple DAA combinations, including NS5A inhibitor + protease inhibitor + SOF or NS5A inhibitor + protease inhibitor + dasabuvir. The SHARED database also contained a small percentage of participants who were treated with older regimens, including the combinations of boceprevir or telaprevir with pegylated interferon and ribavirin; these regimens are no longer recommended for treating HCV. The linkage of these sequences with detailed treatment and demographic information provides an excellent opportunity to characterize RAS and monitor their prevalence and distribution in different parts of the world.

## 4. Key Findings and Presentations

Since the first data merger, SHARED members have presented at numerous international, national, and local meetings held in America (AASLD, CROI, and CLM), Europe (EASL, IWOHD, and ECCMID), and Asia (APASL). The key findings in these presentations are summarized below:

- Selection of RAS was common following DAA treatment failure. About 80–90% of patients who received an NS5AI- or PI-containing regimen harbored drug-resistant HCV following treatment failure. Resistant HCV variants often had two or more mutations conferring a high level of drug resistance in vitro [22];
- A number of “unusual” GT subtypes were identified in patients who failed NS5AI-containing regimens; these included 1I/g, 2c/i/j/q/, 3b/g/h/k, 4b/f/g/k/n/ns/o/q/r/t/v, and 6e/h/p/q/r/xe. Specifically, GT3b/h and 4r virologic failures were largely over-represented among non-GT3a and non-GT4a/d. Each “unusual” GT subtype harbored multiple NS5A RAS that can contribute to high-level of drug resistance, leading to virologic failure [23,24];
- The majority of the GT3a treatment failure patients had either single or dual RAS containing A30K or Y93H. The frequency of co-selecting Y93H with A30K/R/S/T depended on the treatment received. Failure from GLE–PIB and SOF–velpatasvir –voxilaprevir often resulted in a higher frequency of dual RAS than the first-generation DAAs [25];
- About 13% of the GT1b patients diagnosed using the commercial genotyping assays turned out to be GT3 based on the HCV sequences. The misdiagnosed patients were often treated with inappropriate regimens, resulting in virologic failure and RAS selection [26]. HCV sequencing is the method of choice for determining GT subtypes, drug resistance characterization and viral transmission;
- A web-based application, HCV ReCall, which automatically processed and interpreted Sanger HCV sequence data was developed and made freely available to the public. This application generated a summary report containing HCV genotypes, RAS relative to the prototype references, relative peak heights of the RAS mixtures, quality scores for sequencing primers, and alerts for potential contamination among samples. This open-source program is available at https://hcvshared.hcvdb.ubc.ca (accessed on 21 July 2021) [27].

To provide broader access to the SHARED data, we are in the process of setting up an interactive data visualization platform, HCV Nextstrain, adapted from GISAID, which also provides the platform for SARS-CoV2 [28]. HCV Nextstrain integrates the phylogenomic data with the geographic information collected by SHARED. Each HCV sequence is linked with epidemiological, clinical data, DAA regimens, and treatment history information. We have incorporated filters to identify samples with clinically relevant RAS, the number of RAS, and RAS patterns to facilitate RAS characterization. Users can interrogate the distribution of resistant HCV in subgroups of interest with these filters, e.g., RAS distribution in cirrhotic patients treated with LDV–SOF in Italy. The platform can resolve phylogenetic relatedness among samples within a city, e.g., HCV sequences from virologic failures in Sydney, NSW, Australia. With detailed epidemiological and risk factor information, HCV Nextstrain can potentially be used to track resistant HCV transmission networks. A preview version of HCV Nextstrain is available at https://hcvnextstrain.hcvdb.ubc.ca (accessed on 21 July 2021).

## 5. Strength and Limitations of the SHARED Dataset

SHARED offers a unique opportunity to bring together researchers and physicians across disciplines to explore new hypotheses, investigate research questions, compare cross-cohorts, exchange methodologies, and collaborate in complex projects. Our data came from diverse cohorts of investigational studies and local clinics in multiple countries. The comprehensive data linkage and HCV sequences in real-world settings offer an excellent resource for academic and public health research. The SHARED dataset also provides sufficient power to address relatively rare events, such as RAS characteristics in “unusual” genotypes, novel RAS, compensatory mutations, and RAS characteristics following salvage regimens. As data accumulate, our database can provide comprehensive surveillance of the global HCV resistance landscape and transmission networks.

There are also many limitations and challenges. By nature of the real-world data collection, there are significant variabilities among participating laboratories in data interpretation, disease diagnosis, and sequencing. Nevertheless, this challenge provides us an opportunity to leverage each other’s expertise to come up with a workable system. The current dataset comprises mainly GTs 1, 3, and 4, and there are only a handful of sequences from GTs 2, 5, and 6. The paucity of information from these GTs significantly hampers our understanding of drug resistance in resource-limiting countries. RAS characterization relies heavily on in vitro drug susceptibility data. Currently, we depend on published information for data analysis and interpretation for most known RAS; however, in vitro data for the new RAS are not available, presenting a significant challenge to determine their biological relevance. Additionally, the incomplete behavioral and exposure data presents another challenge to construct an adequate surveillance network for viral transmission. Lastly, the recent pandemic has practically halted the SHARED collaboration, as many of us have shifted our research focus to COVID-19. All of these deficiencies underscore the pressing need to call for collective efforts across disciplines and organizations. To map out the global HCV resistance landscape accurately, we need participation from countries enriched with non-GT1a/1b strains and clinics and laboratories with retreatment data. To fill the data gaps, such as drug susceptibility for novel RAS, we need funding support from governments and funding agencies. Finally, to attain HCV transmission surveillance, we need to efficiently integrate epidemiological data with genomic information through collaboration with health agencies. While we continue to address these unmet needs, we invite everyone with data and an interest in HCV to contribute to SHARED. We also encourage students, postdoctoral fellows, staff scientists, and investigators to use the existing SHARED data for their research.

## 6. How to Join SHARED?

SHARED is open to all scientists and clinicians interested in bringing in data, scientific expertise, or financial support for HCV research. For researchers interested in contributing data, please contact Dr. Anita Howe (anita.howe@bccdc.ca) or any SHARED member; a copy of the institutional review board statement and approval number for the studies will be required to initiate the data-sharing agreement. For more information about SHARED, please visit https://hcvdb.med.ubc.ca (accessed on 21 July 2021).

## 7. SHARED Collaborators

Christoph Sarrazin. University Hospital Frankfurt, Frankfurt, Germany.

Beat Müllhaupt. University Hospital Zürich, Zürich, Switzerland.

Julian Schulze zur Wiesch. University Medical Center Hamburg-Eppendorf, Hamburg, Germany.

Peter Buggisch. Institute for Interdisciplinary Medicine IFI, Hamburg, Germany.

Christoph Neumann-Haefelin. University of Freiburg, Freiburg, Germany.

Thomas Berg. University Hospital Leipzig, Leipzig, Germany.

Christoph P. Berg. University of Tübingen, Tübingen, Germany.

Jörn M. Schattenberg. University Medical Center of the Johannes Gutenberg-University, Mainz, Germany.

Christophe Moreno. CUB Hôpital Erasme, Belgium.

Rudolf Stauber. Medical University of Graz, Graz, Austria.

Andrew Lloyd. The Kirby Institute, UNSW, Sydney, New South Wales, Australia.

Gregory Dore. The Kirby Institute, UNSW Sydney, Sydney, Australia.

Gail Matthews. The Kirby Institute, UNSW Sydney, Sydney, Australia.

Tanya Applegate. The Kirby Institute, UNSW Sydney, Sydney, Australia.

Josep Quer. Vall d’Hebron Institut de Recerca (VHIR), Barcelona, Spain.

Juan Ignacio. Instituto de Salud Carlos III, Madrid, Spain.

Damir Garcia-Cehic, Vall d’Hebron Hospital Universitari, Barcelona, Spain.

Josep Gregori. Universitat Autònoma de Barcelona, Barcelona, Spain.

Francisco Rodriguez-Frias. Vall d’Hebron Institut de Recerca (VHIR), Barcelona, Spain.

Ariadna Rando. Vall d’Hebron Hospital Universitari, Barcelona, Spain.

Murat Sayan. Kocaeli University, Kocaeli, Turkey and Near East University, Northern Cyprus, Turkey.

Mario Angelico. Hepatology Unit, University Hospital of Rome Tor Vergata, Rome, Italy.

Massimo Andreoni. Infectious Diseases, University Hospital of Rome Tor Vergata, Rome, Italy.

Valeria Cento. Department of Oncology and Hemato-oncology, Università degli Studi di Milano, Milan, Italy.

Nicola Coppola. University of Campania “L. Vanvitelli”, Napoli, Italy.

Antonio Craxì. Gastroenterology, “P. Giaccone” University Hospital, Palermo, Italy.

Velia Chiara Di Maio. Department of Experimental Medicine, University of Rome Tor Vergata, Rome, Italy.

Stefania Paolucci. Molecular Virology Unit, Microbiology and Virology Department, IRCCS Policlinic Foundation San Matteo, Pavia, Italy.

Giustino Parruti. Infectious Disease Unit, Pescara General Hospital, Pescara, Italy.

Carlo Federico Perno. Unit of Microbiology and Diagnostic Immunology, Bambino Gesù Children’s Hospital, IRCCS Rome, Italy.

Elisabetta Teti. Infectious Diseases, University Hospital of Rome Tor Vergata, Rome, Italy.

Vironet C. HCV Virology Italian Resistance Network Group, https://www.vironetc.org/ (accessed on 21 July 2021).

Midori Kjellin. Uppsala University Hospital, Uppsala, Sweden.

Anders Lannergård. Uppsala University Hospital, Uppsala, Sweden.

Hege Kileng. University Hospital of North Norway, Tromsø, Norway.

Ann-Sofi Duberg. Örebro University, Örebro, Sweden.

Soo Aleman. Karolinska University Hospital, Stockholm, Sweden.

Tore Gutteberg. University Hospital of North Norway, Tromsø, Norway.

Slim Fourati. Centre National de Réference des Hépatites B, C et delta, Créteil, France.

Alexandre Soulier. French National Agency for Research on AIDS and viral Hepatitis, Paris, France.

Aurélie Gourgeon. French National Agency for Research on AIDS and viral Hepatitis, Paris, France.

Stephane Chevaliez. French National Agency for Research on AIDS and viral Hepatitis, Paris, France.

Stanislas Pol. French National Agency for Research on AIDS and viral Hepatitis, Paris, France.

Fabrice Carrat. French National Agency for Research on AIDS and viral Hepatitis, Paris, France.

Dominique Salmon. Paris Descartes University, Paris, France.

Javier Alejandro Sfalcin. Laboratorio CIBIC, Rosario, Argentina.

Fay Fabián Fernando. Laboratorio CIBIC, Rosario, Argentina.

Rolf Kaiser. University Hospital Cologne, Cologne, Germany.

Elena Knopes. University Hospital Cologne, Cologne, Germany.

Perpetua Gomes. Centro Hospitalar Lisboa Ocidental, Hospital Egas Moniz, Lisbon and CiiEM, Almada, Portugal.

Rob de Kneght. Erasmus University Medical Center, Rotterdam, Netherlands.

Bart Rijnders. Erasmus University Medical Center, Rotterdam, Netherlands.

Mario Poljak. University of Ljubljana, Slovenia.

Maja Lunar. University of Ljubljana, Slovenia.

Orna Mor. Sheba Medical Center, Ramat-Gan, Israel.

Rafael Usubillaga. Paris Descartes University, Paris, France.

Carole Seguin_Devaux. Luxembourg Institute of Health, Luxembourg, Luxembourg.

Enoch Tay. Westmead Institute for Medical Research, New South Wales, Australia.

Caroline Wilson. Westmead Institute for Medical Research, New South Wales, Australia.

Dao Sen Wang. University of Sydney and WIMR, New South Wales, Australia.

Jacob George. University of Sydney and Westmead Hospital, New South Wales, Australia.

Jen Kok. NSW Health Pathology, ICPMR, Westmead, New South Wales, Australia.

Ana Belén Pérez. Instituto Maimónides de Investigación Biomédica de Córdoba (IMIBIC). Córdoba, Spain.

Natalia Chueca. University Hospital San Cecilio. Instituto de Investigacion Ibs.Granada, Granada, Spain.

Miguel García-Deltoro. Hospital General de Valencia, Valencia, Spain.

Ana María Martínez-Sapiña. Hosputal Miguel Servet, Zaragoza, Spain.

María Magdalena Lara-Pérez. Hospital Nuestra Señora de la Candelaria, Tenerife, Spain.

Silvia García-Bujalance. University Hospital La Paz, Madrid, Spain.

Teresa Aldámiz-Echevarría. Hospital Gregorio Marañón, Madrid, Spain.

Francisco Jesús Vera-Méndez. University Hospital Santa Lucía, Cartagena, Spain.

Juan Antonio Pineda. University Hospital Nuestra Señora de Valme, Sevilla, Spain.

Marta Casado. Complejo Hospitalario Torrecárdenas, Almería, Spain.

Juan Manuel Pascasio. University Hospital Virgen del Rocío, Sevilla, Spain.

Javier Salmerón. University Hospital San Cecilio Granada, Granada, Spain.

Juan Carlos Alados-Arboledas. University Hospital Jerez, Cadiz, Spain.

Antonio Poyato. University Hospital Reina Sofía, Córdoba, Spain.

Francisco Téllez. Hospital Puerto Real, Cádiz, Spain.

Antonio Rivero-Juárez. University Hospital Reina Sofía. IMIBIC. Universidad de Córdoba. Córdoba, Spain.

Dolores Merino. Complejo Hospitalario de Huelva, Huelva, Spain.

María Jesús Vivancos-Gallego. University Hospital Ramón y Cajal, Madrid, Spain.

José Miguel Rosales-Zábal. Hospital Costa del Sol, Marbella, Spain.

María Dolores Ocete. Hospital General de Valencia, Valencia, Spain.

Miguel Ángel Simón. Hospital Miguel Servet, Zaragoza, Spain.

Pilar Rincón. University Hospital Nuestra Señora de Valme, Sevilla, Spain.

Sergi Reus. Hospital General de Alicante, Alicante, Spain.

Alberto De la Iglesia. Complejo Hospitalario de Huelva, Huelva, Spain.

Isabel García-Arata. Hospital Universitario de Fuenlabrada, Fuenlabrada, Madrid, Spain.

Miguel Jiménez. Hospital Regional de Málaga, Málaga, Spain.

Fernando Jiménez. Complejo Hospitalario de Huelva, Huelva, Spain.

José Hernández-Quero. University Hospital San Cecilio Granada, Granada, Spain.

Carlos Galera. University Hospital Virgen de la Arrixaca, El Palmar, Murcia, Spain.

Mohamed Omar Balghata. Complejo Hospitalario de Jaén, Jaén, Spain.

Joaquín Primo. Hospital de Sagunto, Sagunto, Valencia, Spain.

Mar Masiá. Hospital General de Elche, Elche, Alicante, Spain.

Nuria Espinosa. University Hospital Virgen del Rocío, Sevilla, Spain.

Marcial Delgado. Hospital Regional de Málaga, Málaga, Spain.

Miguel Ángel von-Wichmann. University Hospital Donostia, Donostia, Spain.

Antonio Collado. Complejo Hospitalario Torrecárdenas, Almería, Spain.

Jesús Santos. University Hospital Virgen de la Victoria, Málaga, Spain.

Carlos Mínguez. Castellón II—Penitenciary Institution; Albocásser, Castellón de la Plana, Spain.

Felícitas Díaz-Flores. Hospital Universitario de Canarias, Santa Cruz de Tenerife, Canary Islands, Spain.

Elisa Fernández. Hospital de Poniente, El Ejido, Almería, Spain.

Enrique Bernal. Hospital General Reina Sofía, Murcia, Spain.

José De Juan. Penitenciary Institution, Córdoba, Spain.

José Joaquín Antón. Penitenciary Institution; Albolote, Granada, Spain.

Mónica Vélez. Hospital General de La Palma, Santa Cruz de Tenerife, Canary Islands, Spain.

Antonio Aguilera. Complejo Hospitalario Universitario de Santiago de Compostela, Santiago de Compostela, Spain.

Daniel Navarro. Complejo Hospitalario Universitario de Santiago de Compostela, Santiago de Compostela, Spain.

Juan Ignacio Arenas. University Hospital Donostia, San Sebastian, Spain.

Clotilde Fernández. University Hospital Puerta del Mar, Cádiz, Spain.

María Dolores Espinosa. University Hospital Virgen de las Nieves, Granada, Spain.

María José Ríos. University Hospital Virgen Macarena, Sevilla, Spain.

Roberto Alonso. University Hospital Gregorio Marañón, Madrid, Spain.

Carmen Hidalgo. University Hospital Virgen de las Nieves, Granada, Spain.

Rosario Hernández. University Hospital Torrevieja, Torrevieja, Alicante, Spain.

María Jesús Téllez. Hospital Clínico San Carlos, Madrid, Spain.

Francisco Javier Rodríguez. Hospital General Reina Sofía, Murcia, Spain.

Pedro Antequera. University Hospital José María Morales Meseguer, Murcia, Spain.

Cristina Delgado. Hospital Alto Guadalquivir, Andújar, Jaén, Spain.

Patricia Martín. Hospital de Denia, Denia, Alicante, Spain.

Javier Crespo. University Hospital Marqués de Valdecilla, Santander, Spain.

Berta Becerril. Hospital Punta de Europa, Algeciras, Cádiz, Spain.

Oscar Pérez. Hospital de Castellón de la Plana, Castellón de la Plana, Spain.

Antonio García-Herola. Hospital Marina Baixa, Vilajoyosa, Alicante, Spain.

José Montero. University Hospital Puerto Real, Puerto Real, Cádiz, Spain.

Carolina Freyre. University Hospital Puerto Real, Puerto Real, Cádiz, Spain.

Concepción Grau. Hospital Vega Baja, Orihuela, Alicante, Spain.

Adolfo de Salazar. University Hospital San Cecilio. Instituto de Investigacion Ibs.Granada, Granada, Spain.

Joaquin Cabezas. Marqués de Valdecilla University Hospital, Santander, Spain.

Miguel Jimenez. Hospital Regional de Málaga, Málaga, Spain.

Manuel Alberto Macias Rodriguez. Hospital Universitario Puerta del Mar, Cádiz, Spain.

Cristina Quilez. Hospital Marina Baixa, Alicante, Spain.

Maria Rodriguez Pardo. Hospital Universitario Puerta del Mar, Cádiz, Spain.

Leopoldo Muñoz-Medina. University Hospital San Cecilio Granada, Granada, Spain.

Blanca Figueruela. Hospital Universitario Virgen de Valme, Sevilla, Spain.

Ana Fuentes. University Hospital San Cecilio. Instituto de Investigacion Ibs. Granada, Granada, Spain.

## Figures and Tables

**Table 1:** SHARED cohort participant characteristics.

Characteristics	Total
Number of Patients, *n*	4911
Male sex, *n* (%)	3643 (74%)
Age in 2021, median (IQR)	56 (48–63)
Ethnicity, *n*	832
caucasian, *n* (%)	751 (90%)
black, *n* (%)	20 (2%)
other, *n* (%)	61 (7%)
Illicit Drug use, *n*	1500
injection drug use, *n* (%)	887 (59%)
non-injection drug use, *n* (%)	382 (25%)
Sexual orientation, *n*	902
heterosexual, *n* (%)	528 (59%)
homosexual, *n* (%)	343 (38%)
bisexual, *n* (%)	29 (3%)
Coinfection, *n*	2819
HIV-HCV, *n* (%)	750 (27%)
HBV-HCV, *n* (%)	103 (4%)
Cirrhosis, *n*	2464
yes, *n* (%)	1012 (41%)
Genotype *, *n*	4911
GT1a, *n* (%)	1754 (36%)
GT1b, *n* (%)	1285 (26%)
GT1-other, *n* (%)	33 (1%)
GT 2, *n* (%)	147 (3%)
GT3, *n* (%)	1395 (28%)
GT4, *n* (%)	276 (6%)
GT5, *n* (%)	2 (0.04%)
GT6, *n* (%)	18 (0.4%)
GT8, *n* (%)	1 (0.02%)
Treatment history, *n*	3195
treatment naïve, *n* (%)	2315 (72%)
treatment experienced, *n* (%)	880 (28%)
prior PEG/RBV, *n* (%)	463 (53%)
prior DAA, *n* (%)	141 (16%)
unknown, *n* (%)	276 (31%)
Treatment, *n*	3951
NS5AI + NI, *n* (%)	2203 (56%)
NS5AI + PI, *n* (%)	770 (19%)
PI + NI, *n* (%)	153 (4%)
NS5AI + PI + NI or NNI, *n* (%)	504 (13%)
other, *n* (%)	321 (8%)
Treatment Response to DAA, *n*	3354
sustained viral response	1342 (40%)
virologic failure	2012 (60%)
HCV sequences, *n*	10,332
NS3	2772 (27%)
NS5A	4640 (45%)
NS5B	2472 (24%)
Core -E1-E2	448 (4%)

* Genotypes were derived from the HCV NS5A, NS3, or NS5B sequences. IQR, interquartile range; DAA, direct-acting antivirals; NS5AI, NS5A-inhibitor-containing regimens; PI, protease-inhibitor-containing regimens, NI, nucleoside (sofosbuvir)-containing regimens; NNI, non-nucleoside (dasabuvir)-containing regimens; other, pegylated interferon +/− ribavirin +/− DAA including boceprevir and telaprevir.
